# Locomotive Syndrome Stage 1 Predicts Significant Worsening of Future Motor Performance: The Prospective Yakumo Study

**DOI:** 10.1155/2019/1970645

**Published:** 2019-10-03

**Authors:** Kazuyoshi Kobayashi, Shiro Imagama, Kei Ando, Masaaki Machino, Satoshi Tanaka, Masayoshi Morozumi, Shunsuke Kanbara, Sadayuki Ito, Taro Inoue, Naoki Ishiguro, Yukiharu Hasegawa

**Affiliations:** ^1^Department of Orthopaedic Surgery, Nagoya University Graduate School of Medicine, Aichi, Japan; ^2^Department of Rehabilitation, Kansai University of Welfare Science, Osaka, Japan

## Abstract

**Purpose:**

Aging of society has increased the need for prolongation of a healthy lifespan through maintenance of physical function. Prediction of future physical function may be possible by screening for stage 1 locomotive syndrome (LS). In this prospective study, we examined the influence of LS stage 1 at baseline (2011) on physical performance after 5 years (2016) in a community-dwelling cohort.

**Methods:**

The participants were elderly adults aged >40 years who attended public health checkups as part of the Yakumo Study. LS screening in 2011 and 2016 was performed using the 25-question geriatric locomotive function scale (GLFS-25), the stand-up test, and the two-step test. LS of stage 1 or 2 was defined if the participant met the criteria in any of the three tests. Participants not meeting LS criteria were defined as the no risk group. Physical performance tests (10 m gait time, back muscle strength, 3 m TUG, and maximum stride) were also performed in 2011 and 2016.

**Results:**

A total of 113 subjects (49 males, 64 females; average age 65.0 years) were followed from 2011 to 2016. At baseline, 73 (65%) had no risk, 29 (25%) had stage 1 LS, and 11 (10%) had stage 2 LS. Five years later, 51 (45%) had no risk, 45 (40%) had stage 1 LS, and 17 (15%) had stage 2 LS. Of the 73 subjects with no risk at baseline, 23 (32%) had stage 1 LS and 1 (1%) had stage 2 LS after 5 years. The baseline stage 1 LS group had significantly worse physical performance after 5 years, compared to the baseline no risk group (*p* < 0.05).

**Conclusions:**

This longitudinal study showed that stage 1 LS screening is important for prevention of motor dysfunction in middle-aged and elderly people.

## 1. Introduction

With the current aging of society, health problems of the elderly are important issues in Japan. The aging of the population has led to increased prevalences of various diseases. Therefore, there is a need for prolongation of healthy activities of daily living (ADL) in elderly people, in the interests of the people themselves and of the government. This situation places a focus on physical function, which is strongly associated with ADL and quality of life (QOL) in the elderly population [1–4].

In 2007, the Japanese Orthopaedic Association (JOA) proposed the concept of “locomotive syndrome” (LS), as a condition in people with musculoskeletal disease who are highly likely to require future nursing care [5, 6]. People with LS have significantly lower QOL [7], and prevention of LS has long been advocated for maintaining and improving physical function of middle-aged and elderly people [6, 8–11]. To evaluate the risk of LS, the JOA proposed the following three tests: the two-step test, the stand-up test, and the 25-question geriatric locomotive function scale (GLFS-25) [12]. LS is categorized into stages 1 and 2, and the results of these tests allow mobility and the LS stage to be determined. Stage 1 is a preliminary LS stage that particularly indicates that movement function has begun to decline, and there are likely to be more people in this stage than in stage 2.

Motor performance in LS has been widely studied [13–19], but there have been no prospective studies of future motor performance in stage 1 LS. The “Yakumo study” includes a physical examination in general screening of a residential cohort, which facilitates a cross-sectional examination of physical ability in middle-aged and elderly people. The current prospective study took advantage of this situation to examine the influence of LS stage 1 on physical performance after 5 years in community-dwelling people.

## 2. Methods

### 2.1. Subjects

The participants were Japanese elderly adults who attended the annual public health checkup provided by the local government in Yakumo, Japan, in 2011. We have collected epidemiological data at these annual health checkups since 1983, as the basis of the Yakumo Study. Yakumo is located in Hokkaido, in the north of Japan, and has a population of 17,000 people, of which 28% are elderly (over 65 years old). Yakumo is in a relatively rural area, and many people work in the agriculture and fishery industries. The checkup includes an orthopedic assessment and measurements of physical function, in addition to internal medical examinations and psychological testing [1, 2, 8, 9].

For the current study, which started in 2011, we examined LS by investigating physical function, spinal and joint diseases, and osteoporosis and provided guidance on exercise. Since 2011, we have added completion of the GLFS-25 and four physical performance tests: 10 m gait time, back muscle strength, 3 m timed-up-and-go (3 m TUG), and maximum stride. Age, gender, body mass index (BMI), and bone mineral density (BMD) were also recorded. The inclusion criteria were Japanese males and females aged >40 years who underwent these tests (which are described in more detail below) during the health checkup. Individuals were excluded if they had severe walking or standing disabilities or dysfunction of the central or peripheral nervous system. All physical measurements were made by 6 orthopedic surgeons. The study protocol was approved by our University Committee on Ethics in Human Research. All participants provided written informed consent, and the study protocol was approved by the Institutional Review Board of our University Graduate School of Medicine. The study was carried out in accordance with the principles of the Declaration of Helsinki.

### 2.2. Physical Performance

BMD was measured ultrasonically in the calcaneus using a bone densitometer (A1000 Insight, Lunar Corp., Madison, WI, USA), and the percent of the young adult mean (%YAM) was determined. Diagnosis of osteoporosis was based on criteria of the Japanese Society for Bone and Mineral Research [20] and defined as %YAM < 70% in the calcaneus. The 10 m gait time was measured to evaluate mobility, as the time required to complete a 10 m straight course at the fastest pace possible for each subject. Back muscle strength was examined as the maximal isometric strength of the trunk muscles measured in a standing posture with 30° lumbar flexion using a digital back muscle strength meter (T.K.K.5102, Takei Co., Japan) [1]. The average force from two trials was recorded, and the maximum strength in each trial was measured. The 3 m TUG test was used to measure the time for a subject to rise from a standard chair (46 cm seat height), walk a distance of 3 m, turn around, walk back to the chair, and sit down [21]. Subjects performed the test twice, both at maximum pace, and the mean time was used for analysis. Maximum stride length was measured in a standing position. Subjects placed their right foot forward as far as possible and then brought their left foot up to the right foot without support. The maneuver was then repeated with the left foot stepping forward first. The test was performed twice, and the average step length divided by the height of the subject was used as the maximum stride length for analysis. The % change over 5 years for each physical performance variable was calculated as follows: (value after 5 years—value at baseline)/value at baseline.

### 2.3. LS Stage Tests

Three tests were performed according to the JOA guidelines [22]. In the stand-up test, the ability to adopt a single- or double-leg stance from stools that were 40, 30, 20, and 10 cm high was measured by physical therapists. The level of difficulty was defined as 40 < 30 < 20 < 10 cm using both legs <40 < 30 < 20 < 10 cm using one leg. The result is reported as the minimum stool height from which the participant could stand up. The score was graded from no impairment (8 points) to severe impairment (0 points). Scores <4 and <2 were defined as stage 1 and stage 2 LS, respectively.

In the two-step test, the length of two strides from the starting line to the position at the tips of the toes was measured by physical therapists. The score was calculated by normalizing the maximal length of two steps by height. Scores of <1.3 and <1.1 were defined as stage 1 and stage 2 of LS, respectively.

The GLFS-25 is a self-reported comprehensive survey referring to the preceding month [23]. The scale includes 4 questions on pain, 16 on ADL, 3 on social functions, and 2 on mental health status. Each item was graded from no impairment (0 points) to severe impairment (4 points). Total scores >7 and >16 were defined as stage 1 and stage 2 LS, respectively.

In overall assessment of LS, the stage was deemed to be 1 or 2 if the participant met one of the three criteria above. Participants not meeting any LS criteria were defined as the no risk group.

### 2.4. Statistical Analysis

Continuous variables are presented as mean ± standard deviation (SD) and categorical data as numbers (percentage). A Mann-Whitney *U* test or Student's *t*-test was used for comparison of continuous variables between groups, and a *χ*^2^-test or Fisher's exact test was used for categorical data. A post hoc test was performed using a Bonferroni test to assess which group differed significantly from others. All calculations were performed using SPSS ver. 23 (SPSS Inc., Chicago, IL, USA). Values of *p* < 0.05 were considered to be significant in all analyses.

## 3. Results

**Figure 1:**
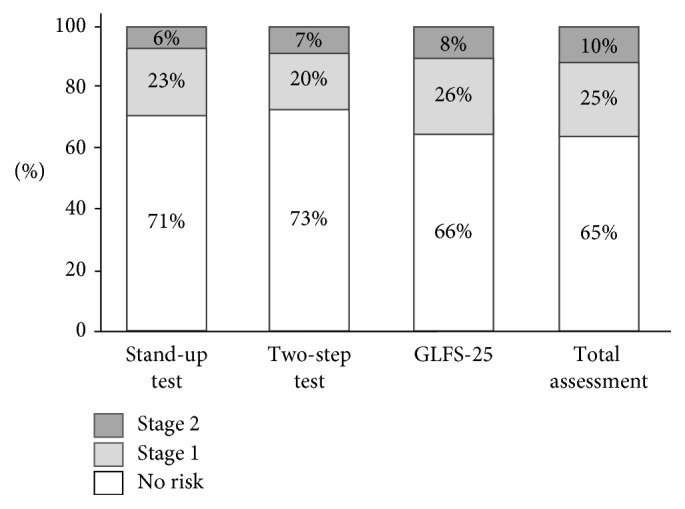
Distribution of risk levels for LS from three criteria (GLFS-25, stand-up test, and two-step test).

**Figure 2:**
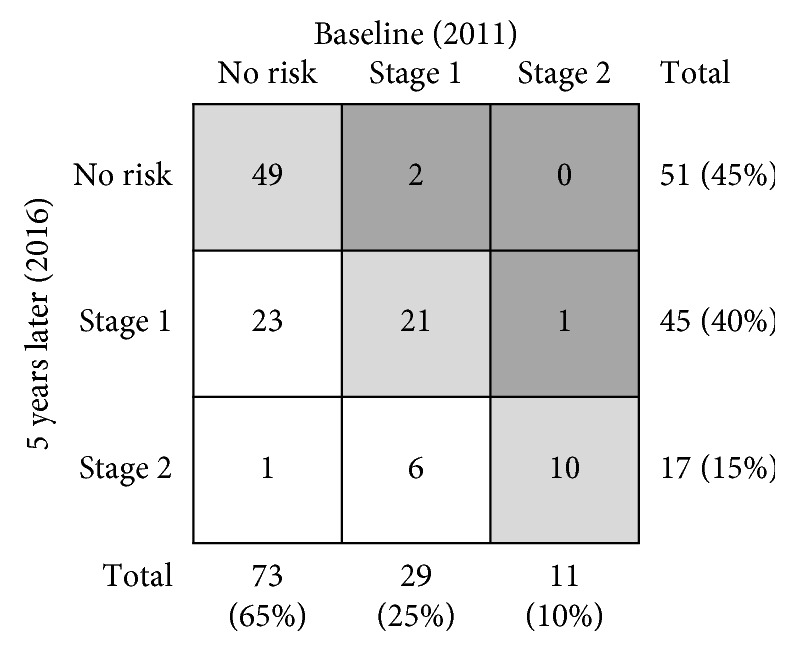
Distribution of risk levels for LS from three tests and the total assessment for all participants at baseline.

A total of 534 subjects participated in the medical examination in 2011, of whom 15 met the exclusion criteria, leaving 519 for baseline evaluation. Of these 519 subjects, 300 could not be followed up for 5 years and we excluded 187 subjects in whom all physical performance tests (10 m gait time, back muscle strength, 3 m TUG, and maximum stride) were not performed. The remaining 113 subjects were included in this study. Demographic and physical performance data at baseline (2011) are shown in Table 1. The 113 subjects had an average age of 65.0 years (range 42–88 years), and 49 were males and 64 were females.  Figure 1 shows the distribution of risk levels of LS from the three tests and the total assessment for all participants. The trend of LS stage from baseline to 5 years later is shown in Figure 2. At baseline, there were 73 subjects (65%) in the no risk group, 29 (25%) with stage 1 LS, and 11 (10%) with stage 2 LS. Five years later, 51 subjects (45%) had no risk, 45 (40%) had stage 1 LS, and 17 (15%) had stage 2 LS. Of the 73 subjects with no risk at baseline, 23 (32%) had stage 1 LS and 1 (1%) had stage 2 LS after 5 years (Figure 2).

**Figure 3:**
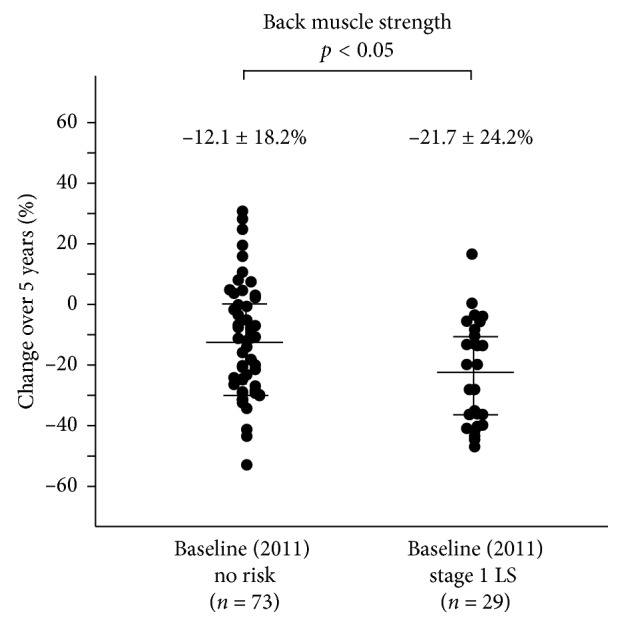
The 5-year reduction in back muscle strength was significantly lower in the baseline no risk group (*n* = 73) compared to the baseline stage 1 LS group (*n* = 29) (−12.1 ± 18.2% vs. −21.7 ± 24.2%; *p* < 0.05).

**Figure 4:**
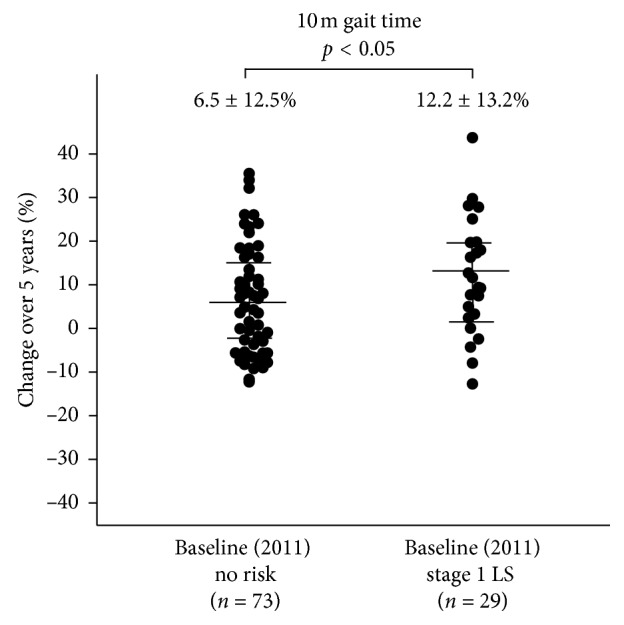
The 5-year increase in 10 m gait time was significantly lower In the baseline no risk group (*n* = 73) compared to the baseline stage 1 LS group (*n* = 29) (6.5 ± 12.5% vs. 12.2 ± 13.2%; *p* < 0.05).

**Figure 5:**
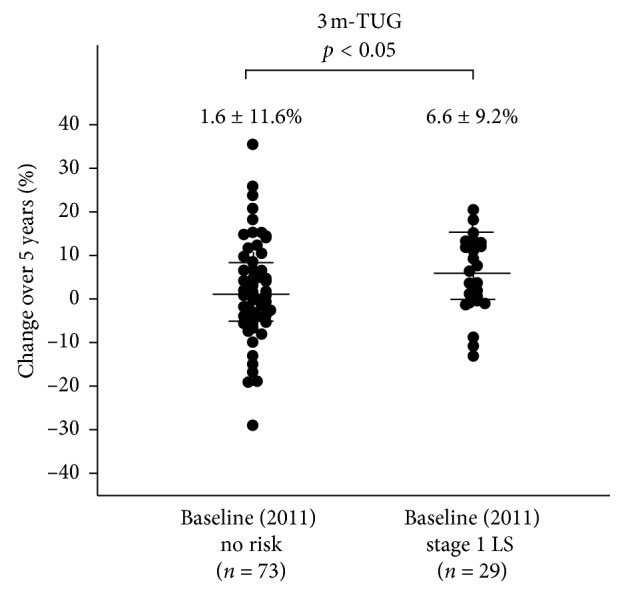
The 5-year increase in 3 m-TUG was significantly lower in the baseline no risk group (*n* = 73) compared to the baseline stage 1 LS group (*n* = 29) (1.6 ± 11.6% vs. 6.6 ± 9.2%; *p* < 0.05).

**Figure 6:**
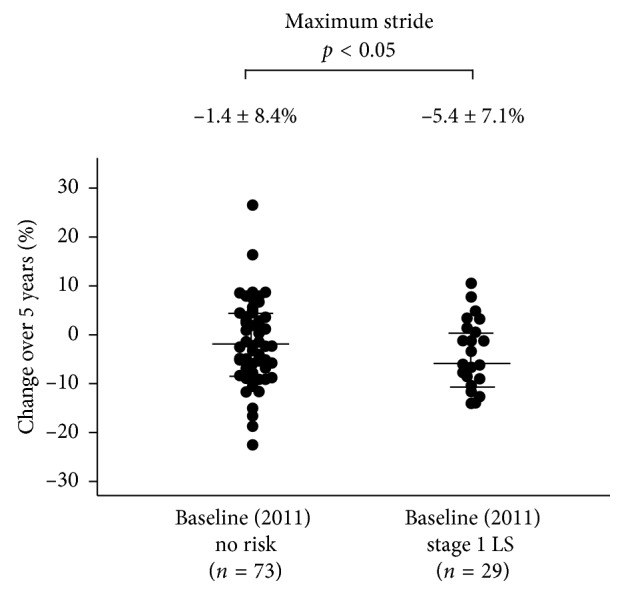
The 5-year reduction in maximum stride was significantly lower in the baseline no risk group (*n* = 73) compared to the baseline stage 1 LS group (*n* = 29) (−1.4 ± 8.4% vs. −5.4 ± 7.1%; *p* < 0.05).

Demographic data at baseline (2011) based on LS stage at baseline are shown in Table 2. Age and the rate of females increased as LS risk stage progressed. Further, the stage 1 LS group was significantly older than the no risk group in both genders and had significant differences in all physical performance tests (10 m gait time, back muscle strength, 3 m TUG, and maximum stride) after 5 years (Table 3). The 5-year changes in these tests all showed significant differences between the baseline no risk and stage 1 LS groups (*p* < 0.05) (Figures 3–6).

## 4. Discussion

There have been several reports on reference values for LS in physical function tests [3–6, 16–19]. Progression of LS, especially in stage 2, limits independence in daily life and could affect physical balance and general conditions [4, 15, 24]. Stage 1 LS is a preliminary stage of LS, in which there may be many more people than in stage 2. However, no prospective study has examined future physical performance in elderly community-dwelling people in LS stage 1. Thus, this is the first prospective study to evaluate motor performance over a 5-year period with a focus onstage 1 LS.

The GLFS-25 was developed as a simple assessment tool for detection of LS [23]. However, JOA criteria recommend use of the stand-up test and two-step test for LS stage evaluation, in addition to the GLFS-25 [22]. Previous studies have only used the GLFS-25 for stage evaluation, but LS includes a decrease in mobility such as standing and walking. Thus, a strength of this study is evaluation of the LS grade using all three JOA criteria, including the stand-up test, two-step test, and GLFS-25. However, we could not clearly conclude which of the criteria had the most influence on motor performance evaluation due to the small sample size. The JOA defines a GLFS-25 score ≥7 as stage 1 LS, in which deterioration of movement is beginning to occur [23]. Interestingly, a score ≥7 is consistent with the GLFS-25 scores of 6.4 for males and 6.8 for females suggested by Kobayashi et al. as thresholds for future LS, which may indicate that stage 1 LS is a risk factor for future stage 2 LS [15].

In our cohort, the rates of older age and female gender increased as the LS risk stage progressed. These variables have also previously been identified as risk factors for LS [11, 25]. Despite the significant difference in age between the no risk and stage 1 LS groups, there were no significant differences between these groups in all motor performance tests at baseline. However, there were significant differences in all tests after 5 years, with significantly greater deterioration of motor performance in the stage 1 LS group compared with the no risk group. Regarding prevention of LS, exercise intervention has been suggested to be effective for reducing deterioration of physical function in elderly people [26–28]. Age at the start of exercise intervention also seems to be important. In our cohort, the average ages were 63.9 years in the no risk group and 65.8 years in the stage 1 LS group, and the cohort also included relatively young people in their 40 s; thus, exercise intervention is likely to be effective in this cohort.

Evaluation of LS is the only one method for screening for musculoskeletal disorders but has an advantage that people with LS also have significantly lower QOL [7]. It has long been advocated that prevention of LS can maintain or improve physical function in middle-aged and elderly people [6, 8–11, 13–19]. In view of our results, measures taken for people in stage 1 LS are likely to be important for future LS prevention, and construction of an early exercise program for LS stage 1 subjects may have a preventive effect on LS.

The current study has some limitations. First, the number of participants who we were able to follow for 5 years was relatively small. Second, performance tests such as 10 m gait time, 3 m TUG, and maximum stride reflect motor performance and mobility of the lower limbs, but none are effectively improved by musculoskeletal intervention. Third, the participants were middle-aged and elderly people who lived in a relatively rural area, in which many had jobs in agriculture or fishing; therefore, the subjects differed from people in an urban environment. Also, the participants attended an annual health examination, which suggests that they may be more health conscious compared to other people. Fourth, we did not examine personal exercise habits, details of medication, and medical comorbidities. The decrease in physical function might also reflect the change in age, and a connection of physical function deterioration with aging cannot be clearly excluded. However, this study is the first to evaluate physical performance prospectively over 5 years with a focus on LS stage 1 in community-dwelling people. LS is generally recognized as stage 2, and stage 1 is treated as a preliminary step. Therefore, recognition of LS stage 1 is likely to be effective for future prevention of LS stage 2. A further strength of the study is that we performed an extensive set of physical measurement that is associated with QOL or ADL. However, further examinations of future physical performance are needed from the perspective of LS grade at baseline.

## 5. Conclusion

In this longitudinal study in a cohort of community-dwelling people, future physical performance and its rate of change over 5 years differed significantly between the no risk and stage 1 LS groups at baseline. These results suggest that people with LS of stage 1 are likely to have significant deterioration in future motor performance. Therefore, for prevention of motor dysfunction in middle-aged and elderly people, LS stage 1 screening is important using the GLFS-25, stand-up test, and two step test, which can be relatively easily performed.

## Figures and Tables

**Table 1:** Demographic data at baseline (2011) in patients (*n* = 113) who were followed for 5 years.

Variable at baseline	Total (*n* = 113)	Male (*n* = 49)	Female (*n* = 64)
Age (years)	63.8 ± 8.5	65.0 ± 7.7	63.0 ± 9.0
≤49	9 (8%)	3 (6%)	6 (9%)
50–59	26 (23%)	10 (20%)	16 (25%)
60–69	48 (42%)	20 (41%)	28 (44%)
≥70	30 (27%)	16 (33%)	14 (22%)
Height (cm)	158.5 ± 8.1	165.6 ± 9.2	152.8 ± 7.2
Body weight (kg)	59.5 ± 9.1	66.1 ± 8.3	54.5 ± 7.2
Body fat percentage (%)	27.4	25.1	29.3
Body mass index (kg/m^2^)	23.6 ± 2.9	24.0 ± 2.5	23.3 ± 3.1
Bone mineral density (%YAM)	81.8 ± 16.6	85.4 ± 18.8	79.7 ± 14.8
Osteoporosis	21 (19%)	7 (14%)	14 (22%)
GLFS-25	7.4 ± 7.1	6.7 ± 7.6	7.9 ± 6.3
Stand-up test (%)^†^	42.9 ± 12.6	47.8 ± 15.2	39.1 ± 10.4
Two-step test (cm)	111.3 ± 18.1	120.6 ± 19.4	104.3 ± 17.3

Values are expressed as mean ± standard error or as a number (percentage). YAM: young adult mean; GLFS-25: 25-question geriatric locomotive function scale. ^†^Number of subjects who could stand up on one leg (right or left) from a height of 40 cm.

**Table 2:** Demographic data at baseline (2011) as a function of LS stage at baseline (2011).

Variable at baseline	Baseline (2011)			Anova *p* value	Post hoc test^1^
	No risk^a^ (*n* = 73)	Stage 1^b^ (*n* = 29)	Stage 2^c^ (*n* = 11)		
Age (years)	61.8 ± 7.4	66.3 ± 8.1	70.9 ± 6.9	<0.05	*a* < *b* < *c*
Female (*n*)	56% (41)	59% (17)	73% (8)	<0.05	a, *b* < *c*
Body mass index (kg/m^2^)	23.3 ± 2.9	23.7 ± 2.8	25.4 ± 2.5	<0.05	a, *b* < *c*
Bone mineral density (%YAM)	82.6 ± 16.8	80.9 ± 17.2	75.2 ± 8.5	<0.05	a, *b* < *c*

Values are expressed as mean ± standard error or as a number (percentage). YAM: Young adult mean. ^1^Post hoc test with Bonferroni correction.

**Table 3:** Physical performance 5 years later (2016) based on LS risk stage in males and females.

Variable	Male			Female		
	Baseline no risk (*n* = 32)	Baseline stage 1 (*n* = 12)	*p* value	Baseline no risk (*n* = 41)	Baseline stage 1 (*n* = 17)	*p* value
Age	62.9 ± 6.8	67.5 ± 9.4	<0.05^*∗*^	60.9 ± 7.6	65.6 ± 7.2	<0.05^*∗*^
5 years later (2016)						
10 m gait time (s)	5.0 ± 0.6	6.0 ± 0.8	<0.01^*∗∗*^	5.2 ± 0.7	6.0 ± 0.5	<0.01^*∗∗*^
Back muscle strength (kg)	90.9 ± 26.2	72.5 ± 14.8	<0.01^*∗∗*^	55.5 ± 14.0	44.4 ± 17.5	<0.05^*∗*^
3 m TUG (s)	5.9 ± 0.6	6.9 ± 0.9	<0.01^*∗∗*^	6.0 ± 0.7	6.8 ± 0.5	<0.01^*∗∗*^
Maximum stride (%)	77.0 ± 7.5	69.3 ± 5.8	<0.01^*∗∗*^	76.8 ± 4.7	71.6 ± 6.0	<0.05^*∗*^

^*∗*^ *p* < 0.05; ^*∗∗*^*p* < 0.01. 3 m TUG: 3 m timed-up-and-go.

## Data Availability

All data referred to in the study are included in the manuscript.
